# Characterization and Anti-Allergic Activity of Exopolysaccharides from a New Probiotic *Lacticaseibacillus casei* LZ9183

**DOI:** 10.3390/foods15162845

**Published:** 2026-08-14

**Authors:** Wanting Li, Yan Liu, Jiahao Zhou, Jie Luo, Chengguo Liu, Xia Tang, Xiankang Fan, Hui Zhou

**Affiliations:** 1 College of Food Science and Technology, Hunan Agricultural University, Changsha 410128, China; 17873529348@163.com (W.L.);; 2 Royal Group Hunan Ushi Dairy Co., Ltd., Changsha 410600, China

**Keywords:** lactic acid bacteria exopolysaccharides, antimicrobial activity, immunological activity, anti-allergic

## Abstract

Lactic acid bacteria exopolysaccharides (LAB EPS) have garnered significant interest owing to their beneficial biological properties and safety. This study aimed to screen LAB EPS with comprehensive biological activities and anti-allergic potential from four high-yield strains, isolate and characterize their homogeneous polysaccharide fractions, and preliminarily explore their anti-allergic mechanism. Through in vitro evaluation of antibacterial, hyaluronidase inhibitory, antioxidant and immunomodulatory activities, *Lacticaseibacillus casei* LZ9183 EPS (183 EPS) was identified as the optimal candidate with the most superior comprehensive performance. Further purification via DEAE-52 anion exchange chromatography and Sephadex G-100 gel filtration chromatography yielded two homogeneous polysaccharide fractions, LZEPS-1 and LZEPS-2. Structural characterization results showed that LZEPS-1 (28.73 kDa) was a neutral polysaccharide composed of rhamnose, galactose, glucose and mannose with a total sugar content of 94.39 ± 0.92%, while LZEPS-2 (95.58 kDa) was an acidic polysaccharide containing glucose, galactose, arabinose, mannose, xylose and glucuronic acid with 92.17 ± 1.22% total sugars and 1.72 ± 0.04% protein. Using the RBL-2H3 cell degranulation model, we demonstrated that 183 EPS, LZEPS-1 and LZEPS-2 effectively alleviated IgE-mediated allergic responses by inhibiting the release of β-hexosaminidase and histamine, and modulating the secretion of IL-4 and TNF-α to restore the Th1/Th2 balance. These results propose that LAB EPS could potentially be utilized as natural anti-allergic functional ingredients in the development of functional foods or dietary supplements.

## 1. Introduction

Exopolysaccharides (EPS) are high-molecular-weight polymers produced by microorganisms during growth and metabolism [1]. These biopolymers can be secreted into the extracellular environment or attached to the cell surface, and they are commonly extracted from fermentation broths followed by purification using techniques such as anion exchange chromatography and gel filtration chromatography [2]. Due to their inherent safety and diverse biological activities, lactic acid bacteria (LAB) EPS have garnered considerable research interest [3,4]. LAB EPS exhibit a wide range of functional attributes, including antioxidant, immunomodulatory, prebiotic, antitumor, cholesterol-lowering, and antibiofilm activities [5,6,7,8]. However, the structural characteristics of EPS, such as monosaccharide composition, glycosidic linkages, and molecular weight, vary significantly depending on the bacterial strain, culture conditions, and extraction methods [9]. These structural variations directly determine their biological functions and potential applications. For instance, EPS with higher mannose content or specific α(1 → 6) glycosidic bonds have been associated with enhanced immunomodulatory activity [10,11]. Therefore, the systematic screening and characterization of novel LAB strains with high EPS yield and specific bioactivities are essential for their industrial application in food, pharmaceuticals, and cosmetics.

The immune system plays a critical role in defending the host against pathogens, eliminating abnormal cells, and maintaining physiological homeostasis. A growing body of evidence has demonstrated that various natural substances can enhance immune function. Compared to synthetic immunomodulators, food-derived bioactive components offer superior safety profiles, making them attractive candidates for functional food development. For example, several studies have reported that regular intake of certain probiotics significantly improves immune responses in both animal models and human trials [12,13]. Probiotics, particularly LAB, exert their immunomodulatory effects mainly through their secreted metabolites [14]. Among these metabolites, EPS are increasingly recognized as key bioactive components responsible for many of the probiotic benefits [15]. Previous studies have revealed considerable variability in EPS from different LAB strains in terms of molecular weight, monosaccharide composition, yield, and functional activity. For instance, Gorzelanna et al. [4] demonstrated that EPS from *Lactobacillus paracasei* VL8 activated RAW 264.7 macrophages via the NF-κB and MAPK signalling pathways, while Gao et al. [5] reported that EPS from *Lactobacillus helveticus* LZ-R-5 exhibited potent immunostimulatory activity. Additionally, Li et al. [16] found that EPS from *Lactobacillus plantarum* R315 displayed strong antioxidant and antibacterial properties. These strain-specific differences highlight the necessity of systematic screening and in-depth characterization of EPS-producing LAB strains. Moreover, the current yields of EPS from LAB are often limited, and the diversity of high-yield, high-activity strains remains underexplored, underscoring the urgent need to expand the screening scope.

Despite the growing understanding of LAB EPS bioactivities, their anti-allergic effects and the underlying molecular mechanisms remain poorly understood. Allergic diseases affect over 4 million people worldwide and impose a heavy healthcare burden, yet conventional treatments only provide symptomatic relief and may cause side effects [17]. Therefore, safe and effective natural anti-allergic agents are urgently needed. EPS from certain LAB strains have been shown to modulate immune responses by interacting with pattern recognition receptors such as Toll-like receptors (TLRs) or mannose receptors (CD206), leading to the activation or suppression of downstream signalling cascades [11,18]. However, direct evidence for the anti-allergic activity of LAB EPS is still lacking. The RBL-2H3 cell line, which closely resembles mast cells and basophils, is a well-established in vitro model for evaluating IgE-mediated allergic reactions, including degranulation and the release of allergic mediators such as β-hexosaminidase, histamine, and cytokines [19,20]. To date, no study has systematically investigated the anti-allergic activity of LAB EPS using this model, nor has the structure–activity relationship been elucidated.

In this study, we selected four LAB strains (LZ9089, LZ9Y10, LZ9183, and LZ9285) from a larger pool based on their superior EPS yields (ranging from 567.07 to 726.73 mg/L, as shown in Section 2.2) and distinct taxonomic diversity, and systematically evaluated their comprehensive biological activities (including antibacterial, hyaluronidase inhibitory, antioxidant, and immunomodulatory effects), purified and structurally characterized the EPS from the optimal strain *Lacticaseibacillus casei* LZ9183, and evaluated its anti-allergic potential using the RBL-2H3 cell degranulation model. This work aims to fill the current research gap and provide a scientific basis for the development of LAB EPS as natural anti-allergic functional ingredients in the food and pharmaceutical industries.

## 2. Materials and Methods

### 2.1. Strains and Reagents

The four bacterial strains exhibiting high EPS production were screened and isolated from milk and dairy products obtained from the primary mountain pastures in Hunan, China. These bacterial strains were identified as *Lacticaseibacillus paracasei* LZ9089 and LZ9Y10, *Lacticaseibacillus casei* LZ9183, and *Lactobacillus brevis* LZ9285, as determined in a prior study [21] through Basic Local Alignment Search Tool (BLAST) analysis of 16S rRNA gene sequences from the National Center for Biotechnology Information (NCBI) database. The culture media and the ELISA kit were purchased from America Sigma Aldrich (St. Louis, MO, USA) and Nanjing Boyan Biotechnology Co., Ltd.(Nanjing, Jiangsu, China), respectively, while other reagents used were analytical grade.

The four bacterial strains exhibiting high EPS production were screened and isolated from milk and dairy products obtained from the primary mountain pastures in Hunan, China. These bacterial strains were identified as *Lacticaseibacillus paracasei* LZ9089 and LZ9Y10, *Lacticaseibacillus casei* LZ9183, and *Lactobacillus brevis* LZ9285, as determined in a prior study [21] through Basic Local Alignment Search Tool (BLAST) analysis of 16S rRNA gene sequences from the National Center for Biotechnology Information (NCBI) database. The culture media and the ELISA kit were purchased from America Sigma Aldrich (St. Louis, MO, USA) and Nanjing Boyan Biotechnology Co. (Nanjing, Jiangsu, China), Ltd., respectively, while other reagents used were analytical grade.

### 2.2. Bacterial Strains and Culture Conditions

LAB strains were cultured in MRS broth at 37 °C for 24 h with two successive activations before use. The EPS yields of LZ9089, LZ9Y10, LZ9183, and LZ9285 were 726.73 ± 6.84, 567.07 ± 14.65, 696.65 ± 6.71, and 658.50 ± 9.49 mg/L, respectively. Crude EPS were extracted from fermentation supernatants according to previously reported methods with slight modifications [22], including protein removal, ethanol precipitation, dialysis, and freeze-drying. The obtained crude EPS were designated as Y10 EPS, 89 EPS, 183 EPS, and 285 EPS. EPS solutions were prepared at concentrations ranging from 50 to 1000 μg/mL for subsequent experiments.

### 2.3. Antibacterial Activity Assay

The antibacterial activities of EPS samples against *Escherichia coli*, *Listeria monocytogenes*, and *Staphylococcus aureus* were evaluated using the Oxford cup diffusion method as described by [23], with minor modifications. Penicillin–streptomycin solution served as the positive control. All samples were sterilized through 0.22 μm membrane filtration prior to use. Following incubation at 37 °C, inhibition zone diameters were measured, and inhibition zones ≥ 4 mm were considered indicative of antibacterial activity.

### 2.4. Hyaluronidase Inhibitory Activity

Hyaluronidase inhibitory activity was determined according to the method reported by [24] with slight modifications. EPS samples of different concentrations were incubated with hyaluronidase and sodium hyaluronate substrates under controlled conditions. The absorbance was subsequently measured at 530 nm to evaluate the inhibitory effect of EPS on hyaluronidase activity.

### 2.5. Antioxidant Activity Assays

The antioxidant capacities of EPS samples were assessed by DPPH radical scavenging and hydroxyl radical scavenging assays according to previously described methods with minor modifications [3,25]. Vitamin C (Vc) was used as the positive control. Absorbance values were recorded at 517 nm and 510 nm, respectively, and the radical scavenging activities were calculated using the following equations.

The DPPH radical scavenging rate was calculated as follows:

(1) $$\mathrm{Scavenging}\;\mathrm{rate}\;(\%)\;=\;\left(1\;-\;\frac{A_{1}\;-\;A_{2}}{A_{0}}\right)\;\times \;100\%$$

The hydroxyl radical scavenging rate was calculated according to the following equation:

(2) $$\mathrm{Scavenging}\;\mathrm{rate}\;(\%)=\left(1\;-\;\frac{A_{1}\;-\;A_{2}}{A_{0}}\right)\;\times \;100\%\;$$

where A_1_ represents the absorbance of the sample group, A_2_ represents the absorbance of the control group, and A_0_ represents the absorbance of the blank group.

### 2.6. Immunomodulatory Activity Assay

The immunomodulatory activities of EPS samples were evaluated using RAW 264.7 macrophages cultured in RPMI-1640 medium supplemented with 10% fetal bovine serum and 1% penicillin/streptomycin under a humidified atmosphere containing 5% CO_2_ at 37 °C [26]. EPS concentrations ranged from 50 to 1000 μg/mL, while lipopolysaccharide (LPS) and 5-fluorouracil (5-FU) were employed as positive controls where appropriate. Cell viability was determined using the CCK-8 assay. Nitric oxide (NO) production was quantified using the Griess reaction method, while macrophage phagocytic activity was evaluated by neutral red uptake assay [27]. The NO concentration was calculated according to the standard regression equation Y = 0.0044x + 0.0544 (R^2^ = 0.9996).

Cell viability was calculated using the following equation:

(3) $$\mathrm{Cell}\;\mathrm{viability}\;\left(\%\right)\;=\;\frac{A_{\mathrm{sample}}}{A_{\mathrm{blank}}}\;\times \;100\%\;$$

Macrophage phagocytic activity was calculated as follows:

(4) $$\mathrm{Phagocytic}\;\mathrm{activity}\;\left(\%\right)\;=\;\frac{A_{\mathrm{sample}}}{A_{\mathrm{blank}}}\;\times \;100\%$$

### 2.7. Purification and Structural Characterization of EPS

As 183 EPS exhibited comparatively superior bioactivities, further purification was performed using DEAE-52 anion-exchange chromatography followed by Sephadex G-100 gel filtration chromatography to obtain two purified fractions, namely LZEPS-1 and LZEPS-2 [22]. Briefly, the crude EPS solution was loaded onto a DEAE-52 cellulose column, eluted sequentially with distilled water and a stepwise NaCl gradient, and the main fractions were collected and further purified on a Sephadex G-100 gel filtration column [28,29,30]. Total carbohydrate content was determined using the phenol–sulphuric acid method, while protein content was measured by the Coomassie Brilliant Blue G-250 assay [28,29]. Ultraviolet spectroscopy and Fourier transform infrared spectroscopy (FT-IR) were employed to analyze characteristic absorption peaks and functional group composition. Monosaccharide composition was determined by ion chromatography, whereas molecular weight distribution was analyzed by high-performance gel permeation chromatography (HPGPC) according to previously reported methods [22,30].

### 2.8. Anti-Allergic Activity Assay

The anti-allergic activities of 183 EPS and its purified fractions were investigated using RBL-2H3 cells sensitized with anti-DNP-IgE and stimulated with DNP-BSA to establish an IgE-mediated degranulation model. Briefly, RBL-2H3 cells were seeded in 96-well plates at a density of 1 × 10^5^ cells/well and cultured overnight. The cells were then sensitized with 0.5 μg/mL anti-DNP-IgE for 2 h, washed twice with PBS, and incubated with various concentrations of EPS samples (50, 250, and 500 μg/mL) for 1 h. Subsequently, the cells were stimulated with 20 ng/mL DNP-BSA for 30 min to induce degranulation. Tifenprofen fumarate was employed as the positive control. β-Hexosaminidase (β-HEX) release was determined according to the method described by Ma et al. [20] with slight modifications. Histamine (HIS), interleukin-4 (IL-4), and tumour necrosis factor-α (TNF-α) levels in culture supernatants were quantified using commercial ELISA kits in accordance with the manufacturer’s instructions.

The β-HEX release rate was calculated using the following equation:

(5) $$\beta \text{-}\mathrm{HEX}\;\mathrm{release}\;(\%)\;=\left(1\;-\;\frac{A_{L\;\mathrm{group}}\;-{\;A}_{N\;\mathrm{group}}}{A_{M\;\mathrm{group}}\;-\;A_{N\;\mathrm{group}}}\right)\;\times \;100\%\;$$

where L group represents the EPS treatment group, M group represents the model group, and N group represents the normal control group.

### 2.9. Statistical Analysis

All experiments were independently performed at least three times, and the results are presented as mean ± standard deviation (SD). Statistical analyses were conducted using SPSS Statistics 21 software. Differences among groups were analyzed by one-way analysis of variance (ANOVA) followed by least significant difference (LSD) multiple comparison tests. Statistical significance was considered at *p* < 0.05.

## 3. Results and Discussion

### 3.1. In Vitro Biological Activities of Crude EPS

#### 3.1.1. Antimicrobial Effect of Crude EPS

**Figure 1:**
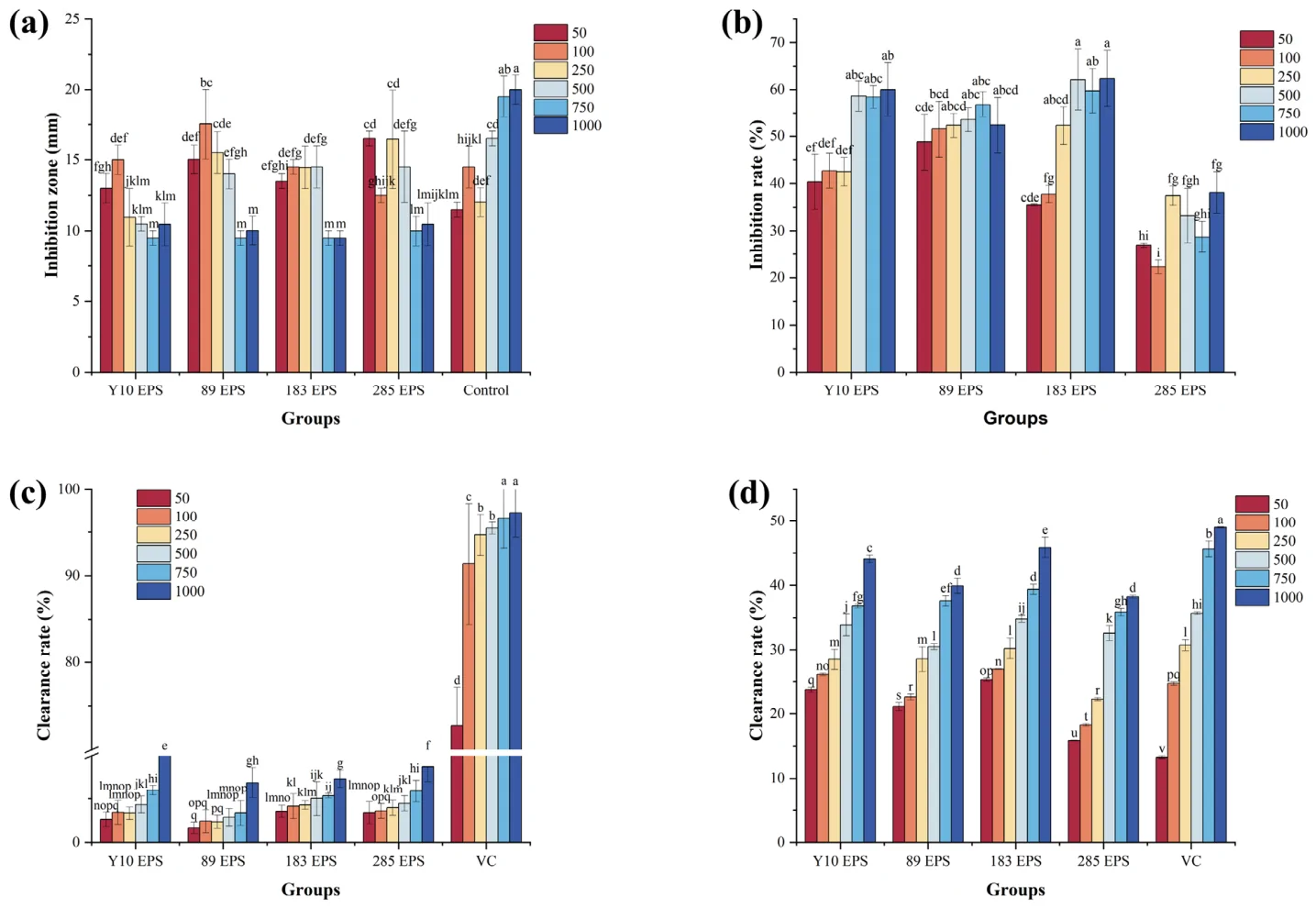
Antimicrobial effects of LAB EPS (**a**). The values are the means ± SD. Positive control: penicillin–streptomycin solution. Hyaluronidase inhibitory effects of LAB EPS (**b**). Antioxidant activities of LAB EPS. (**c**) DPPH free radical scavenging ability. (**d**) Hydroxyl free radical scavenging ability. Different letters indicate significant differences (*p* < 0.05).

The antibacterial activities of crude EPS obtained from four high-yield LAB strains were evaluated against three representative foodborne pathogens, namely *Escherichia coli*, *Staphylococcus aureus*, and *Listeria monocytogenes.* As shown in Figure 1a, all four EPS fractions exhibited significant inhibitory activity against *E. coli* (*p* < 0.05), whereas no evident inhibition was observed against the Gram-positive strains. At 100 μg/mL, 89 EPS displayed the strongest antibacterial activity, producing an inhibition zone diameter of 17.54 ± 2.53 mm, which was significantly greater than that of the penicillin–streptomycin control (*p* < 0.05). Moreover, 89 EPS, 183 EPS, and 285 EPS exhibited stronger antibacterial effects than previously reported EPS derived from *Lactiplantibacillus plantarum*, which demonstrated inhibition zones of approximately 12 mm at 300 μg/mL by Rajoka et al. [31]. Notably, cross-study comparison should be approached with caution, as variations in experimental setups (e.g., strain origin, culture conditions, and assay protocols) may influence the observed antibacterial activities. The superior antibacterial efficacy observed at lower concentrations suggests that the EPS produced in the present study possesses enhanced antimicrobial potency. Differences in antibacterial activity among LAB EPS are generally associated with variations in molecular architecture, glycosidic linkage configuration, molecular weight distribution, and functional group composition [32]. EPS containing uronic acid residues or carboxyl groups may exhibit stronger antimicrobial activity due to increased hydrogen-bonding interactions with bacterial cell envelopes [2]. Furthermore, the selective inhibition of *E. coli* observed in this study may be associated with preferential interactions between EPS and lipopolysaccharides located within the outer membrane of Gram-negative bacteria [33]. In contrast, the thick peptidoglycan layer of Gram-positive bacteria may restrict EPS penetration and reduce susceptibility. However, these proposed mechanisms are speculative and require further experimental validation (e.g., through zeta potential or binding assays) in future studies. Nonetheless, these findings indicate that LAB-derived EPS possess potential application as natural antibacterial agents in food preservation systems [3].

#### 3.1.2. Hyaluronidase Inhibitory Activity of Crude EPS

Hyaluronidase catalyzes the degradation of hyaluronic acid within the extracellular matrix and is closely associated with IgE-mediated allergic reactions [34]. Consequently, inhibition of hyaluronidase activity is widely regarded as an important indicator of anti-allergic potential [24]. As illustrated in Figure 1b, all four EPS fractions exhibited measurable hyaluronidase inhibitory activity. Among them, 183 EPS displayed the strongest inhibitory effect, achieving a maximum inhibition rate of 62.16 ± 6.5% at 500 μg/mL, which was significantly higher than the remaining fractions (*p* < 0.05). This inhibitory activity exceeded the values previously reported for probiotic *Lactobacillus* culture supernatants, which generally exhibited inhibition rates below 40% [24]. Nevertheless, it should be noted that the inhibitory activity of probiotic culture supernatants reported in previous studies may not be directly comparable to that of purified EPS in the present study due to differences in sample purity and complexity. The superior anti-hyaluronidase activity of 183 EPS may be associated with its uronic acid content and negatively charged functional groups. Previous investigations have demonstrated that polysaccharides enriched in uronic acid residues possess enhanced affinity for enzyme active sites, thereby suppressing hyaluronidase-mediated hydrolysis [33]. In contrast to earlier studies primarily focusing on intact bacterial cells, the present results directly demonstrate that purified EPS components themselves contribute substantially to anti-allergic bioactivity. Recent investigations have highlighted the capacity of LAB metabolites to regulate allergic inflammation and modulate immune homeostasis [14]. Nevertheless, reports specifically addressing anti-allergic activities of LAB EPS remain scarce. The pronounced inhibitory effect of 183 EPS therefore provides valuable evidence supporting the development of LAB-derived polysaccharides as functional anti-allergic biomolecules.

#### 3.1.3. Antioxidant Activity of Crude EPS

Reactive oxygen species (ROS) contribute significantly to oxidative stress-mediated cellular damage [35]. Consequently, the antioxidant capacity of LAB EPS was evaluated using DPPH and hydroxyl radical scavenging assays. As indicated in Figure 1c, all four EPS fractions exhibited DPPH radical scavenging activity, although their activities were substantially lower than that of the vitamin C control. No significant differences were observed among the EPS samples (*p* > 0.05), indicating relatively limited DPPH scavenging efficacy. In contrast, Figure 1d shows that hydroxyl radical scavenging activity varied significantly among the EPS fractions. At 1000 μg/mL, 183 EPS exhibited the strongest hydroxyl radical scavenging activity (45.87 ± 1.57%), approaching that of the vitamin C control (49.12 ± 0.95%). The scavenging efficiency followed the order: 183 EPS > Y10 EPS > 89 EPS > 285 EPS. The hydroxyl radical scavenging activity of 183 EPS was comparable to that reported for *Lactobacillus rhamnosus* EPS by Wang et al. [36], yet significantly higher than activities reported for many *Lactobacillus plantarum* EPS fractions. However, direct comparisons across different studies are limited by discrepancies in EPS molecular weight, monosaccharide composition, and assay conditions reported in the literature [36]. Such differences may arise from variations in molecular weight distribution, monosaccharide composition, and glycosidic linkage patterns. Previous studies have demonstrated that polysaccharides with abundant reducing termini and electron-donating functional groups exhibit enhanced ROS-scavenging activity owing to improved hydrogen atom transfer capacity [35]. Importantly, the superior antioxidant performance of 183 EPS was later found to correlate with its distinct monosaccharide profile (Section 3.3.3), indicating a strong structure–activity relationship between EPS composition and antioxidant functionality.

#### 3.1.4. Immunomodulatory Activity of Crude EPS

**Figure 2:**
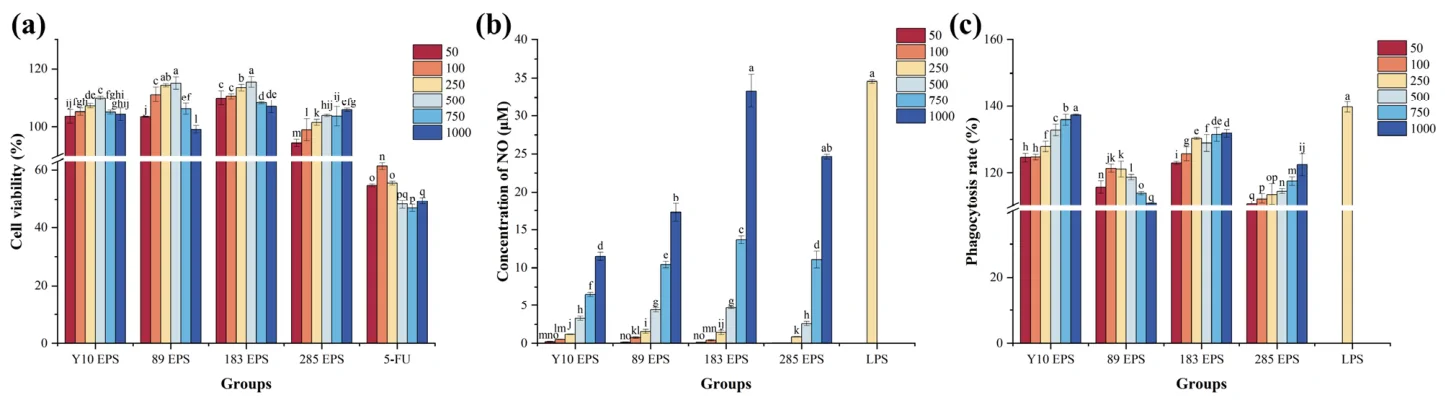
Effects of LAB EPS on the proliferation (**a**), NO production (**b**), and phagocytic activity (**c**) of RAW 264.7 cells. Different letters indicate significant differences (*p* < 0.05).

Macrophages constitute a critical component of innate immunity and play a central role in inflammatory regulation. Therefore, the immunomodulatory effects of LAB EPS were evaluated using RAW264.7 macrophages. According to Figure 2a, Y10 EPS, 89 EPS, and particularly 183 EPS significantly enhanced RAW264.7 cell proliferation within the concentration range of 50–500 μg/mL (*p* < 0.05). At 500 μg/mL, 183 EPS induced the highest proliferation rate (115.62 ± 1.84%), exceeding the activity reported for *Lactobacillus paracasei* VL8 EPS, as reported by Gao et al. [5]. It should be noted that the comparison with the previously reported proliferation activity [5] may be influenced by differences in cell culture conditions, EPS concentrations, and macrophage passage numbers across studies. The enhanced proliferative activity may involve activation of NF-κB and MAPK signalling pathways, which are frequently associated with immunostimulatory polysaccharides [5]. Nitric oxide (NO) production is an established marker of macrophage activation. As observed in Figure 2b, all EPS fractions significantly increased NO secretion in a dose-dependent manner (*p* < 0.05). Among them, 183 EPS induced the highest NO production at 1000 μg/mL. The enhanced NO secretion may be associated with the elevated mannose content subsequently identified in 183 EPS, as mannose-rich polysaccharides can interact with macrophage mannose receptors and activate downstream immune signalling pathways [11]. Phagocytic activity analysis further confirmed the immunostimulatory potential of the EPS fractions (Figure 2c). Y10 EPS and 183 EPS exhibited the strongest stimulatory effects, with phagocytic rates of 137.37 ± 0.23% and 131.87 ± 1.17%, respectively, at 1000 μg/mL. Similar enhancement of macrophage phagocytosis has been reported for EPS derived from *Lacticaseibacillus paracasei* by Liu et al. [29]. Collectively, these findings demonstrate that 183 EPS possesses superior biological functionality, including antioxidant, antibacterial, anti-allergic, and immunomodulatory activities. Consequently, 183 EPS was selected for further purification and structural characterization.

### 3.2. Isolation and Purification of 183 EPS

**Figure 3:**
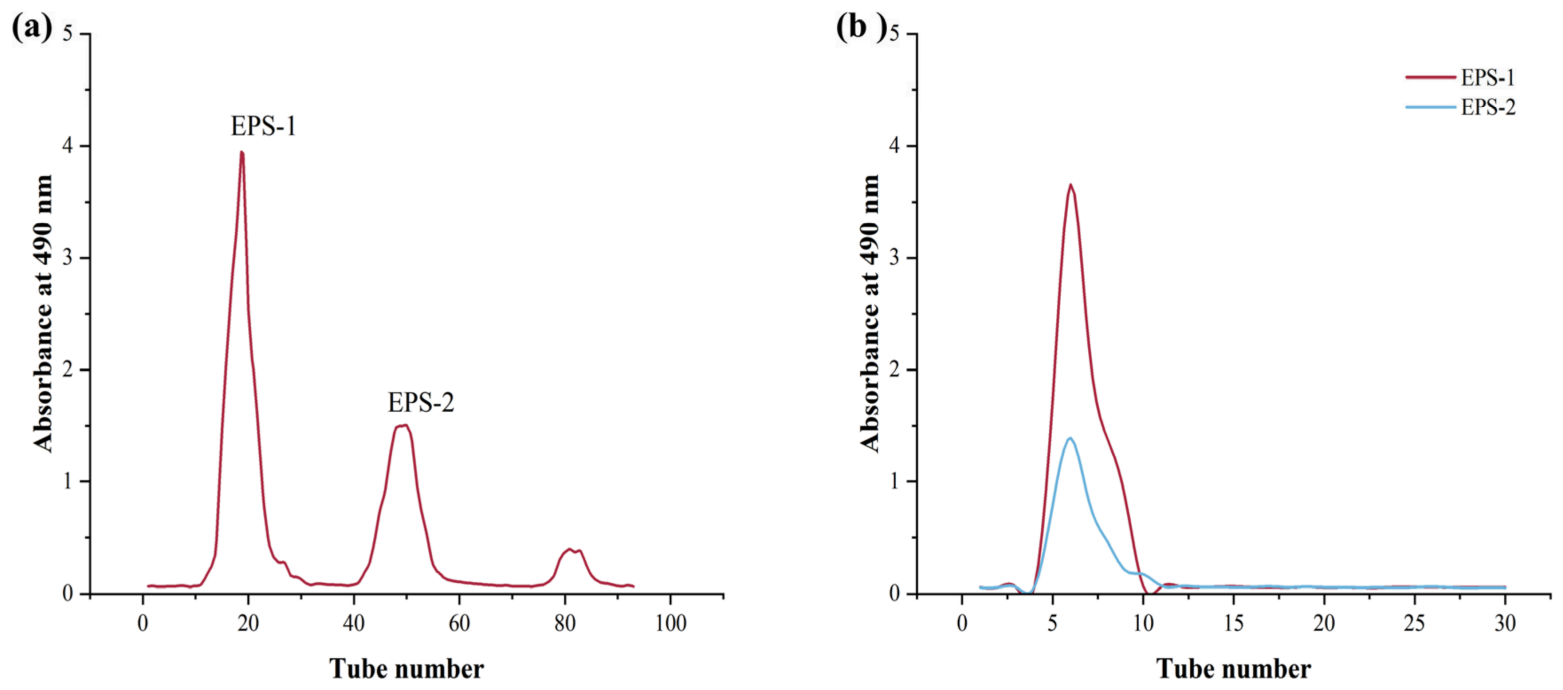
Elution profiles of 183 EPS on a DEAE-Cellulose-52 column (**a**), and elution profiles subsequently obtained using a Sephadex G-100 gel column (**b**).

Anion-exchange chromatography and gel-filtration chromatography were employed to purify 183 EPS. As presented in Figure 3a, DEAE-cellulose-52 chromatography separated 183 EPS into three fractions according to charge distribution and ionic interaction characteristics. EPS-1 was eluted with deionised water, indicating a neutral polysaccharide fraction, whereas EPS-2 was subsequently eluted with 0.1 M NaCl, suggesting the presence of negatively charged acidic substituents, such as uronic acid residues or phosphate groups. Similar elution behaviour has been reported for acidic LAB-derived polysaccharides, in which increased ionic strength was required to disrupt electrostatic interactions between anionic polysaccharides and the DEAE matrix by Zhou et al. [33]. Compared with previously reported LAB EPS that were predominantly neutral glucans, the appearance of an acidic fraction in the present study indicates greater structural heterogeneity of 183 EPS, which may contribute to its superior immunomodulatory and anti-allergic activities observed in Section 3.1. Subsequent purification using Sephadex G-100 gel-filtration chromatography yielded single and symmetrical elution peaks for both fractions (Figure 3b), indicating relatively homogeneous polysaccharide populations with narrow molecular-weight distributions [37]. The absence of overlapping peaks suggested effective removal of residual proteins, nucleic acids, and heterogeneous polysaccharide components during the purification process. Previous studies have demonstrated that polysaccharide homogeneity is closely associated with reproducible biological activity because structural uniformity facilitates more stable receptor–ligand interactions and improves the reliability of structure–activity relationship analyses. Furthermore, the successful isolation of two structurally distinct fractions, namely LZEPS-1 and LZEPS-2, provided an essential basis for subsequent investigations into the influence of molecular weight, monosaccharide composition, and glycosidic linkage patterns on their antioxidant, immunomodulatory, and anti-allergic properties. In particular, the later observation that LZEPS-1 exhibited superior anti-allergic activity suggests that purification-induced enrichment of specific low-molecular-weight glucan fractions may enhance biological functionality through improved solubility, conformational flexibility, and cellular accessibility.

### 3.3. Characterization of 183 EPS

#### 3.3.1. Chemical Composition

The chemical compositions of crude 183 EPS and its purified fractions are presented in Table 1. LZEPS-1 exhibited the highest total carbohydrate content (94.39 ± 0.92%), followed by LZEPS-2 (92.17 ± 1.22%) and crude 183 EPS (87.92 ± 1.74%). Protein was undetectable in LZEPS-1, whereas crude 183 EPS and LZEPS-2 contained 4.94 ± 0.23% and 1.72 ± 0.04% protein, respectively. These results indicated that sequential DEAE-cellulose-52 and Sephadex G-100 chromatography effectively removed proteinaceous impurities and improved polysaccharide purity, consistent with previous reports on LAB-derived EPS purification by Zhu et al. [37]. The high carbohydrate purity and absence of residual protein in LZEPS-1 may be associated with its superior anti-allergic activity observed in Section 3.4, as highly purified polysaccharides generally exhibit enhanced receptor accessibility and improved bioactivity [33].

#### 3.3.2. UV and FT-IR Spectral Analysis

**Figure 4:**
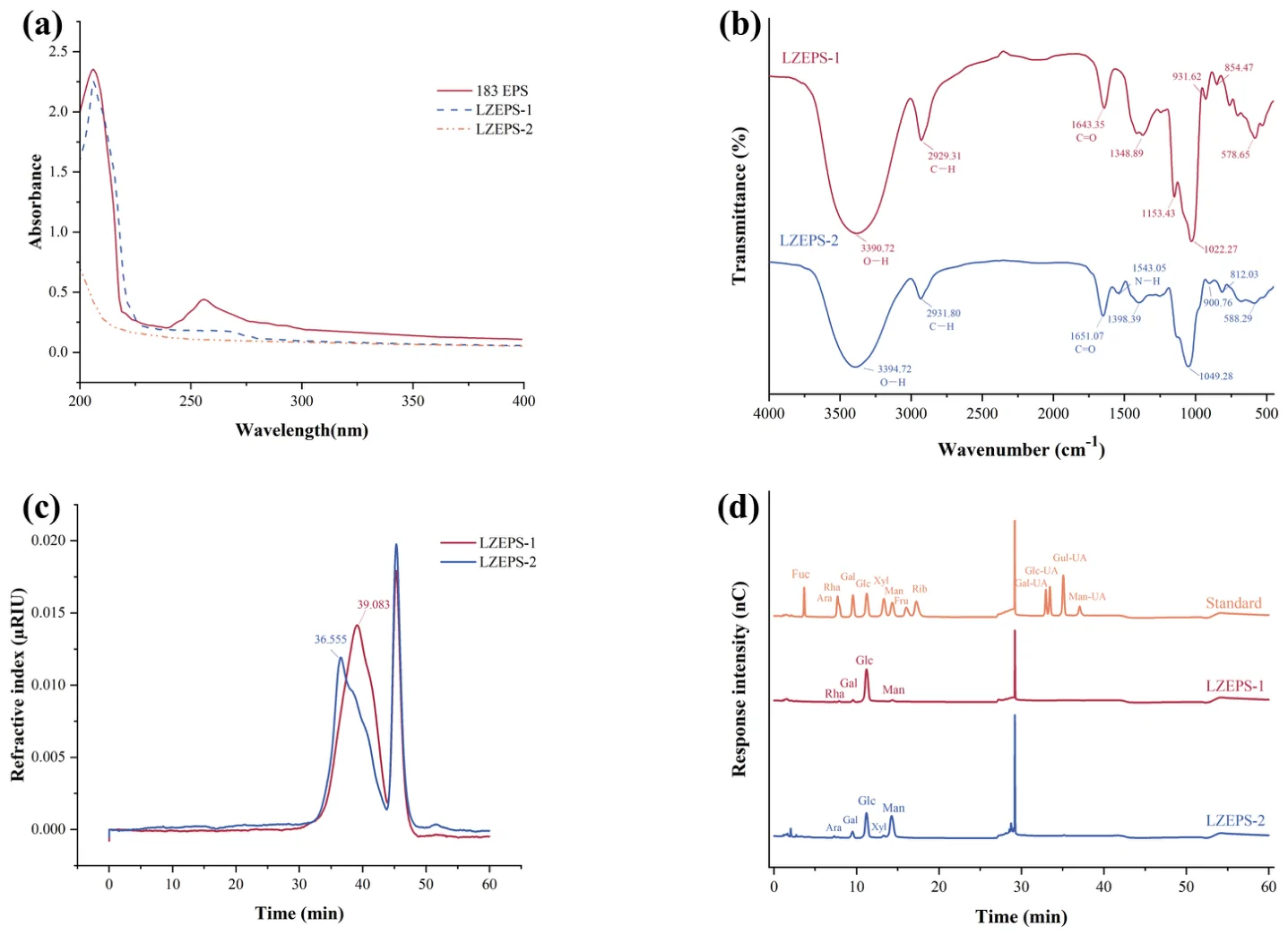
UV–vis spectra of 183 EPS and its purified fractions (**a**), FT-IR spectra of LZEPS-1 and LZEPS-2 (**b**). HPGPC elution profiles of LZEPS-1 and LZEPS-2 (**c**). IC profile of monosaccharide standard, LZEPS-1 and LZEPS-2 (**d**). Note: Fuc: fucose; Ara: arabinose; Rha: rhamnose; Gal: galactose; Glu: glucose; Xyl: xylose; Man: mannose; Fru: fructose; Rib: ribose; Gal-UA: galacturonic acid; Glc-UA: glucuronic acid; Gul-UA: guluronic acid; and Man-UA: mannuronic acid.

The UV spectra of 183 EPS and its purified fractions are shown in Figure 4a. Crude 183 EPS exhibited characteristic absorption peaks at 260–280 nm, indicating the presence of nucleic acids and proteins. In contrast, LZEPS-1 showed no detectable absorption at either 260 or 280 nm, confirming its high purity and the effective removal of protein and nucleic acid contaminants during chromatographic purification. LZEPS-2 displayed a weak absorption peak at 280 nm, consistent with the residual protein detected in the compositional analysis. These findings demonstrated strong agreement between spectroscopic and chemical analyses. FT-IR spectroscopy was subsequently employed to investigate the structural characteristics of the purified polysaccharides (Figure 4b). Both LZEPS-1 and LZEPS-2 exhibited broad absorption bands at 3390.72 cm^−1^ and 3394.72 cm^−1^, respectively, corresponding to O–H stretching vibrations typical of polysaccharides [38]. Absorption peaks at 2929.31 cm^−1^ and 2931.80 cm^−1^ were assigned to C–H stretching vibrations [39]. Strong peaks detected at 1643.35 cm^−1^ and 1651.07 cm^−1^ suggested the presence of carbonyl (C=O) groups and indicated uronic acid residues within both fractions [37]. A weak absorption peak at 1543.05 cm^−1^ was observed only in LZEPS-2, corresponding to N–H bending vibrations of amide groups, further confirming the presence of trace protein residues. Peaks at 1153.43 cm^−1^ and 1022.27 cm^−1^ were attributed to C-O-C and C-O-H stretching vibrations characteristic of pyranose structures. Notably, the absorption region around 1016–1020 cm^−1^ indicated the presence of α-(1 → 6)-glycosidic linkages in LZEPS-1, which are commonly associated with dextran-like glucans [40]. Previous studies have demonstrated that α-(1 → 6)-linked glucans possess greater conformational flexibility and enhanced receptor accessibility, thereby facilitating stronger immunomodulatory activity [10]. This structural feature may therefore contribute to the pronounced immunoregulatory and anti-allergic activities of LZEPS-1 observed in subsequent experiments. In contrast, absorption peaks at 900.76 cm^−1^ and 812.03 cm^−1^ indicated the presence of β-glycosidic linkages in LZEPS-2, consistent with structural characteristics previously reported for bioactive heteropolysaccharides and ulvan-type polysaccharides by Ganesan et al. [41]. β-linked polysaccharides generally exhibit distinct biological functions from α-linked glucans, particularly in anti-inflammatory regulation rather than direct immune stimulation. These structural differences may partly explain the functional divergence observed between LZEPS-1 and LZEPS-2 in the immunological and anti-allergic assays.

#### 3.3.3. Mw and Monosaccharide Composition

The HPGPC chromatograms of purified EPS fractions are presented in Figure 4c. Both LZEPS-1 and LZEPS-2 exhibited single symmetrical peaks, confirming homogeneous polysaccharide characteristics. The molecular weights of LZEPS-1 and LZEPS-2 were determined as 28.73 kDa and 95.58 kDa, respectively, in Table 2. These values are at the lower end of the molecular weight range commonly reported for LAB-derived EPS (10^4^–10^6^ Da) [33]. Variations in molecular weight are closely associated with strain specificity, fermentation conditions and purification procedures. Molecular weight is considered a critical structural determinant governing polysaccharide bioactivity. Previous studies demonstrated that low-molecular-weight polysaccharides possess enhanced aqueous solubility, reduced steric hindrance and improved receptor-binding efficiency, thereby facilitating stronger interactions with membrane-associated immune receptors [42,43]. Accordingly, the relatively low molecular weight of LZEPS-1 may contribute to its superior anti-allergic activity observed in Section 3.4 through enhanced accessibility to FcεRI-associated signalling molecules. In contrast, the higher molecular weight of LZEPS-2 may favour structural stability and prolonged immunomodulatory activity. The monosaccharide compositions of purified EPS fractions were analyzed by ion chromatography, and the chromatographic profiles are depicted in Figure 4d. LZEPS-1 was composed of glucose, rhamnose, galactose, and mannose, with glucose being the predominant monosaccharide (86.32 mol%), whereas LZEPS-2 exhibited a mannose-rich heteropolysaccharide structure, with mannose accounting for 56.72% of the total monosaccharide content. Compared with previously reported LAB-derived EPS mainly composed of glucose and rhamnose [10], the high mannose proportion of LZEPS-2 represented a distinctive structural characteristic. Mannose-rich polysaccharides have been reported to activate macrophages through mannose receptor-mediated NF-κB and MAPK signalling pathways [11], which may explain the enhanced immunomodulatory activity of LZEPS-2 observed in Section 3.1.4. In contrast, the glucose-dominant composition and relatively lower molecular weight of LZEPS-1 may improve molecular flexibility and receptor accessibility, which are structural features commonly associated with enhanced bioactivity in low-molecular-weight polysaccharides [42].

In contrast, LZEPS-1 exhibited a glucose-dominant glucan structure with comparatively lower molecular weight and higher purity. Low-molecular-weight glucans with relatively linear α-linked conformations have been reported to exhibit enhanced receptor accessibility and improved cellular uptake efficiency in allergic and inflammatory models [42]. The predominance of glucose residues and absence of residual protein may therefore contribute to the stronger anti-allergic activity of LZEPS-1 observed in the β-HEX, histamine and cytokine inhibition assays described in Section 3.4. Collectively, the structural analyses demonstrated that LZEPS-1 and LZEPS-2 possess distinct molecular architectures characterized by different molecular weight distributions and monosaccharide compositions, which appear closely associated with their differential immunomodulatory and anti-allergic activities. Nevertheless, the precise glycosidic conformations and higher-order structural characteristics remain unresolved and require further elucidation by NMR spectroscopy and methylation analysis.

### 3.4. Anti-Allergic Effect of EPS

#### 3.4.1. Inhibitory Effects of 183 EPS and Its Purified Fractions on the Release of β-HEX

**Figure 5:**
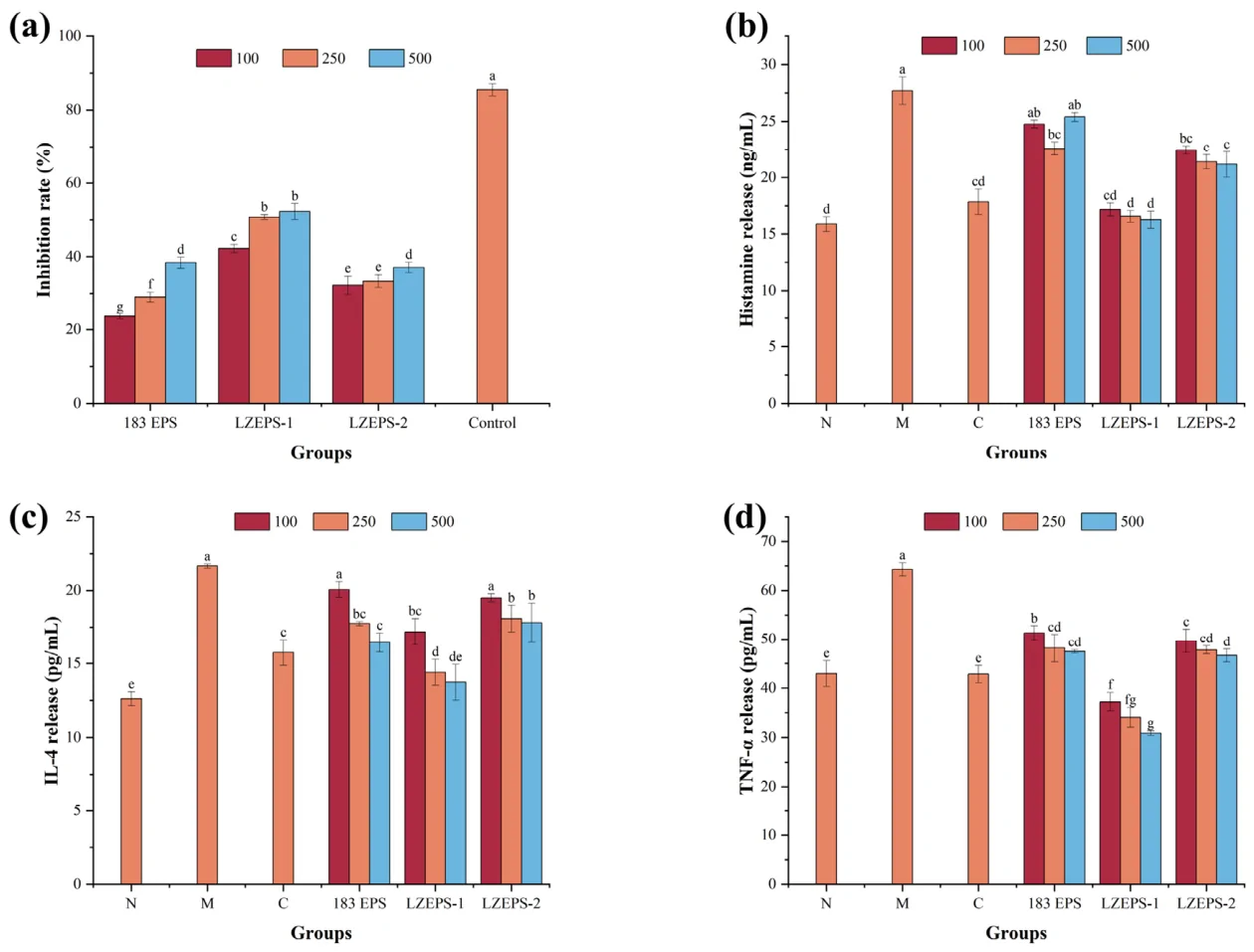
Inhibitory effects of 183 EPS and its purified fractions on the release of β-HEX in RBL-2H3 cells (**a**) Inhibitory effects of 183 EPS and its purified fractions on the release of beta-HEX in RBL-2H3 cells. (**b**) Histamine release (ng/mL). (**c**) IL-4 release (pg/mL). (**d**) TNF-alpha release (pg/mL). N: normal group; M: model group; C: positive control group. Different letters indicate significant differences (*p* < 0.05).

β-HEX release from RBL-2H3 cells was evaluated to assess the anti-allergic activity of 183 EPS and its purified fractions. As revealed in Figure 5a, all EPS fractions significantly inhibited β-HEX release (*p* < 0.05), indicating suppression of mast cell degranulation. Among them, LZEPS-1 exhibited the strongest inhibitory activity, reaching 52.29 ± 2.15% inhibition at 500 μg/mL. This inhibitory effect was markedly higher than that reported for pectin oligosaccharides at the same concentration (~35%) by Ma et al. [20]; nonetheless, it should be noted that the degranulation inhibition activity may vary depending on the structural characteristics of polysaccharides and the specific experimental conditions employed in different studies [20], suggesting superior anti-degranulation activity of LAB-derived EPS. The enhanced activity of LZEPS-1 may be associated with its lower molecular weight and higher polysaccharide purity, which could improve receptor accessibility and facilitate interference with FcεRI-mediated signalling pathways [42,43]. Previous studies have demonstrated that low-molecular-weight polysaccharides possess reduced steric hindrance and enhanced interaction with membrane-associated signalling molecules, thereby improving biological activity. Combined with the hyaluronidase inhibitory activity observed in Figure 1b, these findings indicate that LZEPS-1 possesses pronounced anti-allergic potential through suppression of allergic mediator release.

#### 3.4.2. Effects of 183 EPS and Its Purified Fractions on Histamine, IL-4 and TNF-α

Histamine release is a critical indicator of IgE-mediated mast cell degranulation. As indicated in Figure 5b, LZEPS-1 markedly suppressed histamine secretion in RBL-2H3 cells (*p* < 0.05), reducing histamine levels from 27.71 ± 1.21 ng/mL in the model group to approximately 16 ng/mL, with inhibitory efficacy approaching that of the positive control. This inhibitory effect was stronger than that previously reported for polysaccharides derived from *Lacticaseibacillus paracasei* (~19 ng/mL) by Lee et al. [17], suggesting enhanced suppression of mast cell degranulation. The secretion profiles of IL-4 and TNF-α are presented in Figure 5c and Figure 5d. LZEPS-1 exhibited the strongest inhibitory activity against IL-4, with reductions of 33.55% and 36.64% at 250 and 500 μg/mL, respectively, exceeding the inhibitory capacity reported for *Lactobacillus rhamnosus*-derived EPS in allergic models (~20%) by Liu et al. [44]. It is important to recognize that these comparisons, while encouraging, should be interpreted with caution, as differences in cell culture conditions, sensitization protocols, and cytokine detection methods among studies [17,44] may influence the observed anti-allergic outcomes. In addition, all EPS fractions significantly downregulated TNF-α secretion compared with the model group (*p* < 0.05), whereas LZEPS-1 showed the greatest suppressive effect. The differential anti-allergic activities of LZEPS-1 and LZEPS-2 were closely associated with their structural characteristics described in Section 3.3. The lower molecular weight and glucose-dominant composition of LZEPS-1 may enhance molecular mobility and receptor accessibility, potentially facilitating interactions with FcεRI-associated signalling molecules, including Syk and PLCγ. However, this proposed mechanism requires direct experimental validation in future studies (e.g., via Western blotting or kinase activity assays). Previous studies have demonstrated that low-molecular-weight polysaccharides possess improved cellular accessibility and enhanced immunoregulatory efficacy owing to reduced steric hindrance and increased conformational flexibility [42,43]. Conversely, the mannose-rich heteropolysaccharide structure of LZEPS-2 may preferentially modulate mannose receptor-mediated immune signalling pathways [11]. Collectively, these findings demonstrated that LAB-derived EPS attenuated allergic responses through suppression of β-HEX and histamine release, together with downregulation of IL-4 and TNF-α secretion. The results further support a close structure–activity relationship between molecular weight distribution, monosaccharide composition and the anti-allergic properties of LAB-derived EPS. Despite these promising findings, several limitations should be acknowledged. The anti-allergic effects observed in vitro require further validation in suitable in vivo models, and the proposed mechanisms involving FcεRI-associated signalling molecules need direct experimental verification in future studies [11,42].

## 4. Conclusions

In this study, four high EPS-producing LAB strains were screened, and EPS from Lacticaseibacillus casei LZ9183 was selected for its superior comprehensive bioactivities. Two purified fractions, LZEPS-1 (28.73 kDa, neutral glucan) and LZEPS-2 (95.58 kDa, acidic heteropolysaccharide), were obtained and structurally characterized. Using the RBL-2H3 cell degranulation model, both fractions, particularly the low-molecular-weight glucan LZEPS-1, demonstrated significant anti-allergic activity by inhibiting β-hexosaminidase and histamine release and modulating IL-4 and TNF-α secretion to restore Th1/Th2 balance. These findings highlight the potential of LAB EPS as natural anti-allergic functional ingredients for application in functional foods or pharmaceuticals. For future research, in vivo studies using appropriate animal models are warranted to validate the anti-allergic efficacy and safety of LZEPS-1. Additionally, detailed structural characterization through NMR spectroscopy and methylation analysis should be performed to fully elucidate the glycosidic linkage patterns and higher-order conformations of the purified fractions.

## Figures and Tables

**Table 1:** The components of 183 EPS and its purified fractions.

Components	183 EPS	LZEPS-1	LZEPS-2
Total sugar content (%)	87.92 ± 1.74%	94.39 ± 0.92%	92.17 ± 1.22%
Protein content (%)	4.94 ± 0.23%	-	1.72 ± 0.04%

Note: Values are expressed as mean values (*n* = 3). Different lowercase letters within the same column indicate significant differences (*p* < 0.05).

**Table 2:** The *Mw* and monosaccharide compositions of the purified 183 EPS fractions.

Samples	*Mw* (kDa)	Monosaccharide Compositions (mol %)						
		Ara	Rha	Gal	Glu	Xyl	Man	Glc-UA
LZEPS-1	28.73	0	4.	4.37	86.32	0	4.87	0
LZEPS-2	95.58	0.64	0	8.32	31.67	2.33	56.72	0.32

Note: Values are expressed as mean values (*n* = 3). Different lowercase letters within the same column indicate significant differences (*p* < 0.05).

## Data Availability

The original contributions presented in this study are included in the article. Further inquiries can be directed to the corresponding author.

## References

[1] Angelin J., Kavitha M. (2020). Exopolysaccharides from Probiotic Bacteria and Their Health Potential. Int. J. Biol. Macromol..

[2] Daba G.M., Elnahas M.O., Elkhateeb W.A. (2021). Contributions of Exopolysaccharides from Lactic Acid Bacteria as Biotechnological Tools in Food, Pharmaceutical, and Medical Applications. Int. J. Biol. Macromol..

[3] Hu S.-M., Zhou J.-M., Zhou Q.-Q., Li P., Xie Y.-Y., Zhou T., Gu Q. (2021). Purification, Characterization and Biological Activities of Exopolysaccharides from Lactobacillus Rhamnosus ZFM231 Isolated from Milk. LWT Food Sci. Technol..

[4] Gorzelanna Z., Mamrot A., Bdkowska D., Bubak J., Miszczak M. (2025). Exploring the Potential of Novel Animal-Origin Probiotics as Key Players in One Health: Opportunities and Challenges. Int. J. Mol. Sci..

[5] Gao J., Li Q., Liu Y., Yang B., Sadiqb F.A., Li X., Mi S., Sang Y. (2022). Immunoregulatory Effect of Lactobacillus Paracasei VL8 Exopolysaccharide on RAW264. 7 Cells by NF-κB and MAPK Pathways. J. Funct. Foods.

[6] Saadat Y.R., Khosroushahi A.Y., Gargari B.P. (2019). A Comprehensive Review of Anticancer, Immunomodulatory and Health Beneficial Effects of the Lactic Acid Bacteria Exopolysaccharides. Carbohydr. Polym..

[7] Ye Z.-W., Guo T.-F., Tang C., Yuan Y., Zhao Y., Lu J., Lin J.-F., Guo L.-Q. (2021). Transcriptional Analysis for Cholesterol-Lowering Effects of Marine Lactobacillus Plantarum Lp10 Isolated from Kelp. LWT Food Sci. Technol..

[8] Nguyen P.T., Nguyen H.T. (2024). Antioxidant Property and Structural Characteristics of Exopolysaccharides Derived from Lactiplantibacillus Plantarum VAL6. Discov. Bact..

[9] Shao L.I., Wu Z., Zhang H., Chen W., Ai L., Guo B. (2014). Partial Characterization and Immunostimulatory Activity of Exopolysaccharides from Lactobacillus Rhamnosus KF5. Carbohydr. Polym..

[10] Li S., Huang R., Shah N.P., Tao X., Xiong Y., Wei H. (2014). Antioxidant and Antibacterial Activities of Exopolysaccharides from Bifidobacterium Bifidum WBIN03 and Lactobacillus Plantarum R315. J. Dairy Sci..

[11] Tester R., Al-Ghazzewi F.H. (2017). Role of Glucomannans in Immunology. J. Pharm. Pharm. Sci..

[12] Kim K.-T., Kim J.-W., Kim S.-I., Kim S., Nguyen T.H., Kang C.-H. (2021). Antioxidant and Anti-Inflammatory Effect and Probiotic Properties of Lactic Acid Bacteria Isolated from Canine and Feline Feces. Microorganisms.

[13] Steiner N.C., Lorentz A. (2021). Probiotic Potential of Lactobacillus Species in Allergic Rhinitis. Int. Arch. Allergy Immunol..

[14] Hajavi J., Esmaeili S.-A., Varasteh A.-R., Vazini H., Atabati H., Mardani F., Momtazi-Borojeni A.A., Hashemi M., Sankian M., Sahebkar A. (2019). The Immunomodulatory Role of Probiotics in Allergy Therapy. J. Cell. Physiol..

[15] Zhang K., Liu S., Liang S., Xiang F., Wang X., Lian H., Li B., Liu F. (2024). Exopolysaccharides of Lactic Acid Bacteria: Structure, Biological Activity, Structure-Activity Relationship, and Application in the Food Industry: A Review. Int. J. Biol. Macromol..

[16] Li W., Ji J., Chen X., Jiang M., Rui X., Dong M. (2014). Structural Elucidation and Antioxidant Activities of Exopolysaccharides from Lactobacillus Helveticus MB2-1. Carbohydr. Polym..

[17] Lee J.Y., Kang J.-H., Jung Y.-R., Kang C.-H. (2023). Lactobacillus Gasseri MG4247 and Lacticaseibacillus Paracasei MG4272 and MG4577 Modulate Allergic Inflammatory Response in RAW 264.7 and RBL-2H3 Cells. Probiotics Antimicrob. Proteins.

[18] Rana T.S., Bansode R.R., Williams L.L. (2024). Anti-Allergic and Anti-Inflammatory Signaling Mechanisms of Natural Compounds/Extracts in in Vitro System of RBL-2H3 Cell: A Systematic Review. Cells.

[19] Ahmed I., Ma J., Li Z., Lin H., Xu L., Sun L., Tian S. (2019). Effect of Tyrosinase and Caffeic Acid Crosslinking of Turbot Parvalbumin on the Digestibility, and Release of Mediators and Cytokines from Activated RBL-2H3 Cells. Food Chem..

[20] Ma J., Tong P., Chen Y., Wang Y., Ren H., Gao Z., Yue T., Long F. (2022). The Inhibition of Pectin Oligosaccharides on Degranulation of RBL-2H3 Cells from Apple Pectin with High Hydrostatic Pressure Assisted Enzyme Treatment. Food Chem..

[21] Chen J., Zhou J.-H., Mao Q.-Q., Tang X., Liu C.-G., Zhou H. (2023). Screening and identification of extracellular polysaccharide-producing lactic acid bacteria. Food Mach..

[22] Ji X., Yan Y., Hou C., Shi M., Liu Y. (2020). Structural Characterization of a Galacturonic Acid-Rich Polysaccharide from Ziziphus Jujuba Cv. Muzao. Int. J. Biol. Macromol..

[23] Wu S., Lei W., Zhou H., You S., Wu N., Song Y. (2019). Screening and Identification of Lactic Acid Bacteria with Antibacterial Activity in the Milk of Nanshan Pasture. China Brew..

[24] Lei W., Liu C., Pan L., Peng C., Wang J., Zhou H. (2021). Screening of Probiotic Lactobacilli with Potential Anti-Allergic Activity Based on Hyaluronidase Inhibition and Degranulation of RBL-2H3 Cells in Vitro. LWT Food Sci. Technol..

[25] Shi M.-J., Wei X., Xu J., Chen B.-J., Zhao D.-Y., Cui S., Zhou T. (2017). Carboxymethylated Degraded Polysaccharides from Enteromorpha Prolifera: Preparation and in Vitro Antioxidant Activity. Food Chem..

[26] You X., Li Z., Ma K., Zhang C., Chen X., Wang G., Yang L., Dong M., Rui X., Zhang Q. (2020). Structural Characterization and Immunomodulatory Activity of an Exopolysaccharide Produced by Lactobacillus Helveticus LZ-R-5. Carbohydr. Polym..

[27] Xu W., Fang S., Wang Y., Zhang T., Hu S. (2020). Molecular Mechanisms Associated with Macrophage Activation by Rhizoma Atractylodis Macrocephalae Polysaccharides. Int. J. Biol. Macromol..

[28] Kolsi R.B.A., Salah H.B., Jardak N., Chaaben R., Jribi I., El Feki A., Rebai T., Jamoussi K., Allouche N., Blecker C. (2017). Sulphated Polysaccharide Isolated from Sargassum Vulgare: Characterization and Hypolipidemic Effects. Carbohydr. Polym..

[29] Liu Y.T., Wang J., Ye M., Li J. (2019). In Vitro Hypoglycemic and Hypolipidemic Activities of LEP. Adv. Microbiol..

[30] Wang K., Niu M., Song D., Song X., Zhao J., Wu Y., Lu B., Niu G. (2020). Preparation, Partial Characterization and Biological Activity of Exopolysaccharides Produced from Lactobacillus Fermentum S1. J. Biosci. Bioeng..

[31] Rajoka M.S.R., Mehwish H.M., Zhang H., Ashraf M., Fang H., Zeng X., Wu Y., Khurshid M., Zhao L., He Z. (2020). Antibacterial and Antioxidant Activity of Exopolysaccharide Mediated Silver Nanoparticle Synthesized by Lactobacillus Brevis Isolated from Chinese Koumiss. Colloids Surf. B Biointerfaces.

[32] Silva L.A., Lopes Neto J.H.P., Cardarelli H.R. (2019). Exopolysaccharides Produced by Lactobacillus Plantarum: Technological Properties, Biological Activity, and Potential Application in the Food Industry. Ann. Microbiol..

[33] Zhou Y., Cui Y., Qu X. (2019). Exopolysaccharides of Lactic Acid Bacteria: Structure, Bioactivity and Associations: A Review. Carbohydr. Polym..

[34] Fujitani N., Sakaki S., Yamaguchi Y., Takenaka H. (2001). Inhibitory Effects of Microalgae on the Activation of Hyaluronidase. J. Appl. Phycol..

[35] Andrew M., Jayaraman G. (2020). Structural Features of Microbial Exopolysaccharides in Relation to Their Antioxidant Activity. Carbohydr. Res..

[36] Wang L., Li K., Cui Y., Peng H., Hu Y., Zhu Z. (2023). Preparation, Structural Characterization and Neuroprotective Effects to against H2O2-Induced Oxidative Damage in PC12 Cells of Polysaccharides from Pleurotus Ostreatus. Food Res. Int..

[37] Zhu Z., Chen J., Chen Y., Ma Y., Yang Q., Fan Y., Fu C., Limsila B., Li R., Liao W. (2022). Extraction, Structural Characterization and Antioxidant Activity of Turmeric Polysaccharides. LWT Food Sci. Technol..

[38] Abid Y., Casillo A., Gharsallah H., Joulak I., Lanzetta R., Corsaro M.M., Attia H., Azabou S. (2017). Production and Structural Characterization of Exopolysaccharides from Newly Isolated Probiotic Lactic Acid Bacteria. Int. J. Biol. Macromol..

[39] Gao S., Zong L., Zhang Y., Zhang Y., Guo X., Guo G., Zhao L., Ye F., Fu Y. (2023). Antifungal Pentachloronitrobenzene/Hydroxypropyl-Beta-Cyclodextrin Inclusion Complex Nanofibers by Electrospun with No Polymer: Fabrication and Characterization. J. Clean. Prod..

[40] Ahmed R.Z., Siddiqui K., Arman M., Ahmed N. (2012). Characterization of High Molecular Weight Dextran Produced by Weissella Cibaria CMGDEX3. Carbohydr. Polym..

[41] Ganesan A.R., Shanmugam M., Bhat R. (2018). Producing Novel Edible Films from Semi Refined Carrageenan (SRC) and Ulvan Polysaccharides for Potential Food Applications. Int. J. Biol. Macromol..

[42] Yan J.-K., Wang Y.-Y., Ma H.-L., Wang Z.-B. (2016). Ultrasonic Effects on the Degradation Kinetics, Preliminary Characterization and Antioxidant Activities of Polysaccharides from Phellinus Linteus Mycelia. Ultrason. Sonochem..

[43] Yao Y., Zhu Y., Ren G. (2016). Immunoregulatory Activities of Polysaccharides from Mung Bean. Carbohydr. Polym..

[44] Liu Y., Zhou X., Ye W., Liu Y., Luo J., Tang X., Wang J., Liu C., Zhou H. (2024). Effect of Lactobacillus Rhamnosus LZ260E on Allergic Symptoms and Intestinal Microbiota in β-Lactoglobulin–Sensitized Mice. J. Funct. Foods.

